# Spontaneous Intracranial Hemorrhage Concurrent With Subarachnoid and Subdural Hemorrhages: Report of a Rare Case

**DOI:** 10.7759/cureus.46939

**Published:** 2023-10-13

**Authors:** Mohammed Khaleel I. Kh. Almadhoun, Abdallah Wasel Hattab, Nemer Nedal Alazzeh, Sufyan Taleb Aladwan

**Affiliations:** 1 Medicine and Surgery, Mutah University, Karak, JOR; 2 Neurosurgery, Mutah University, Karak, JOR; 3 Neurosurgery, Thamar University, Amman, JOR; 4 Neurological Surgery, Hashemite University, Amman, JOR

**Keywords:** hypertensive vasculopathy, intracerebral hemorrhage, subdural hemorrhage, subarachnoid hemmorhage, spontaneous intracranial hemorrhage

## Abstract

Spontaneous intracerebral hemorrhage (SICH) is a rare occurrence in the temporal lobe, and its coexistence with other intracranial bleeding types such as subdural hemorrhage (SDH) and subarachnoid hemorrhage (SAH) is infrequently documented. Typically, SICH is managed conservatively without surgical intervention. In this case report, we present an unusual case of SICH in the temporal lobe, characterized by bleeding extending beyond the brain parenchyma into the subarachnoid and subdural spaces. Our approach involved tubular hematoma evacuation (surgical approach).

Literature reports propose the coexistence of SICH, SAH, and SDH, particularly when there is bleeding through the cortical surface that extends into the subdural space. The decision to surgically remove a hematoma in supratentorial ICH remains a subject of debate, as the risks associated with the procedure may outweigh potential benefits in many cases. Surgical intervention is typically reserved for patients with supratentorial ICH causing life-threatening mass effect, with treatment plans tailored based on prognosis assessments with and without surgical intervention. In our patient, craniotomy with tubular evacuation of the hematoma proved effective in alleviating symptoms and preventing life-threatening herniation complications.

## Introduction

Intracerebral hemorrhage, which is the second most common cause of stroke after ischemic stroke is often classified as spontaneous or traumatic. Spontaneous intracerebral hemorrhage (SICH) is characterized by the presence of hemorrhage in the brain parenchyma in the absence of trauma and occurs due to specific etiologies that exclude trauma. SICH accounts for 9-27% of the stroke occurring globally [1]. The most common etiologies include hypertensive vasculopathy, cerebral amyloid angiopathy, and ruptured arterio-venous and other malformations like cavernous malformations [2]. SICH has devastating consequences on patients and their families with a mortality rate of around 40% in the first month and decreased quality of life from the associated morbidities [3]. It is usually seen in the basal ganglia, internal capsule, thalamus, and frontal lobe regions [4]. It is very rarely seen in the temporal lobe. Lobar bleeds account for 10-15% of all SICHs. SICH presence concomitant with other types of intracranial bleeding, subdural hemorrhage (SDH), and subarachnoid hemorrhage (SAH) is rarely reported [5]. SICH is managed expectantly without surgical intervention in most cases. Here we present an interesting case of SICH in the temporal lobe, unusual location, with bleeding not limited to brain parenchyma with extension to subarachnoid and subdural spaces. We managed the case with tubular hematoma evacuation.

## Case presentation

A 67-year-old previously healthy female came to the ER with a sudden onset of severe headache, vomiting, and decreased level of consciousness. The headache was the patient’s worst headache of her life. On admission, the patient had a Glasgow Coma Scale (GCS) of 14/15, pronator drift on the right side, and receptive aphasia. There was no medication or medical history of hypertension, diabetes, or trauma. On examination, her vital signs were as follows: body temperature, 36.7°C; pulse rate, 65 beats/min; and blood pressure, 130/90 mmHg. The results of laboratory tests were within normal limits. Supportive management was started in the patient.

**Figure 1:**
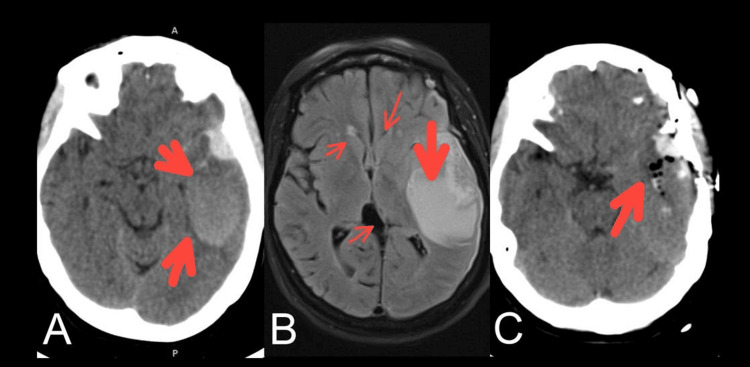
Brain radiological images A: Axial Brain CT scan showing SICH with acute and subacute components, subarachnoid hemorrhage, mass effect on the temporal horn and uncus. B: Brain MRI-Flair sequence showing the thin rim of subdural hematoma and subarachnoid hemorrhage in the Sylvian fissure (midline shift is more appreciated) and edema. C: Postoperative axial Brain CT scan showing SICH evacuation and improvement of mass effect on the uncus. SICH: Spontaneous Intracranial Hemorrhage

On arrival at the ER, a non-contrast computerized tomography (NCCT) scanning of the head was done which showed intraparenchymal bleeding centered in the left Sylvian fissure and temporal lobe causing a local mass effect, sulcal effacement, and 4-mm midline shift to the contralateral side with early herniation in the ipsilateral side as outlined in (Figure 1).

**Figure 2:**
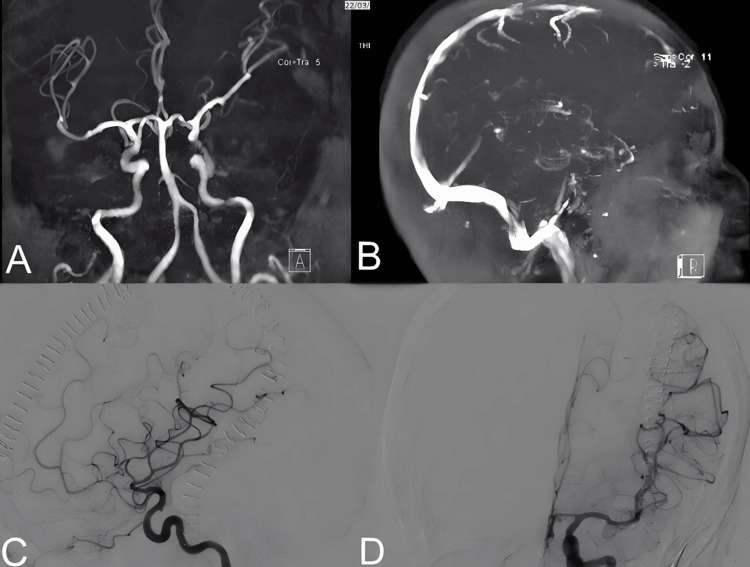
Brain MRA and MRV A: Brain MRA. B: Brain MRV. C: Lateral view DSA. D: Anteroposterior (AP) view DSA showing no evidence of any aneurysms, AVM, fistula, or other vascular pathologies. SICH: Spontaneous Intracranial Hemorrhage; DSA: Digital Subtraction Angiography; FLAIR: Fluid Attenuated Inversion Recovery; MRA: Magnetic Resonance Arteriography; MRV: Magnetic Resonance Venography; AVM: Arteriovenous Malformation

Based on the clinical and NCCT features, the differential diagnoses included: ruptured aneurysm, hemorrhagic brain tumors, or other vascular lesions. To confirm the suspected lesions, a contrasted magnetic resonance imaging of the brain with Magnetic Resonance Arteriography (MRA) and Magnetic Resonance Venography (MRV) followed by a diagnostic angiogram of the internal carotid artery, external carotid artery, and vertebral arteries was performed. However, all the mentioned diagnostic modalities did not show any masses, aneurysms, or arteriovenous malformation (AVM) as evidenced in (Figure 2).

Supportive therapy did not significantly improve the features of raised intracranial pressure (ICP)(vomiting, headache, blurred vision, sleepiness) in our patient. With evident clinical worsening (GCS: 10/15), such as vomiting, headache, blurred vision, sleepiness, and imaging evidence of progressive raised ICP and potential herniation sequelae, immediate neurosurgical consultation was obtained. The neurosurgical team went for craniotomy with tubular evacuation of the hematoma; consequently, the patient improved. There was also improvement seen in the mass effect on uncus as depicted in Figure 1C.

## Discussion

SICH coexisting with subarachnoid and subdural hemorrhage is very rare with only one case reported till now [5]. Based on our recent review of the literature, this would mark the second instance of such a case being documented. Rather there are case reports of SDH combined with SAH but without SICH in the literature [6]. Moreover, this will be the first case report explaining the temporal lobe being the infrequent site for bleeding in SICH.

Less common etiologies for SICH include: hypertensive vasculopathy and cerebral amyloid angiopathy (CAA), AVM, cerebral venous thrombosis, hemorrhagic infarction, bleeding disorders, sickle cell disease, cerebral hyperperfusion syndrome, cerebral vasculitis, Moyamoya disease, mycotic intracranial aneurysm, central nervous system infection, and primary or metastatic tumor. Old age, hypertension, and the use of antiplatelet and anticoagulant therapy are the major risk factors for spontaneous ICH. Other risk factors include obesity, high alcohol intake, race, ethnicity, tobacco use, stimulant drug use, infections, and small vessel vascular disease.

The proposed hypothesis of the presence of concomitant SICH, SAH, and SDH can be found in the literature. SDH and SAH may accompany SICH when there is bleeding through the cortical surface that expands into the subdural space. SICHs involving lobar regions and concomitant SDH have been linked to cerebral amyloid angiopathy [7,8]. SAH caused by a ruptured cerebral aneurysm may extend into the subdural space and this occurs in approximately 0.5% up to 7.9% of cases [9]. Rarely, aneurysm rupture results in discrete SDH without obvious SAH [10]. Rarely, a cortical surface rupture of an arteriovenous malformation, arteriovenous fistula, or cavernous malformation will present as an isolated acute SDH [11]. Stretching of the arachnoid layer, which is attached to the AVM can result in direct bleeding into the subdural region, which is exacerbated by brain movement. Similarly, blood from a pia-arachnoid rupture that is high flow could extravasate into the subdural area. Last but not least, SDH may also be brought on by a ruptured bridge artery, a cortical artery that runs between the cortex and the dura layer [12]. To summarize, there are five mechanisms listed in the literature that can cause SDH combined with SICH and SAH: (I) adhesion of the aneurysm to the arachnoid membrane because of minor successive hemorrhages; (II) extension and then erosion of the aneurysm in the cavernous sinus; (III) laceration of the arachnoid membrane because of high-pressure hemorrhage; (IV) an ICH that tears the brain tissues and possibility of an aneurysm in the subdural carotid artery directly resulting in a hematoma [13,14]

Patients who have severe brain atrophy are more likely to develop SDH due to rupture of the bridging veins between the brain's dural membrane and cortical surface. A history of chronic alcohol misuse, being older, and having experienced a traumatic brain injury are all risk factors for developing cerebral atrophy. In those patients, insignificant head trauma or even a pure whiplash injury without a physical impact may result in an SDH [15]. Senile cortical atrophy due to SDH and CAA leading to lobar hemorrhage (SICH) is possible in our patient. However, it is also possible that the hematoma or surrounding edema might have obscured and confused the evaluation of any potential structural pathology. After the bleeding and edema have subsided in these situations, delayed imaging may be used to find patients who have underlying structural abnormalities and are at a greater risk of experiencing SICH recurrence.

Management of SICH requires immediate triage with aggressive care in the ICU or stroke unit with a focus on the management of acute bleeding, anticoagulant reversal, blood pressure management, and managing hemorrhagic expansion with intra-cranial pressure management. ICP monitoring with clinical features, invasive or imaging modalities is very crucial in the management of SICH. In most patients general and medical therapy for ICP management is sufficient. Emergent surgical intervention may be needed in some patients of SICH with clinical evidence of or concerning imaging features for rapidly progressive neurological dysfunction due to raised ICP. This was true for our case.

Surgery to remove a hematoma in the case of supratentorial ICH is debatable since, in many instances, the risks associated with the procedure may outweigh any potential advantages (STICH). The patient subset that might benefit from surgical treatment has not been firmly established [16]. The surgical intervention is reserved in most of the centers for patients who have supratentorial ICH that poses a life-threatening mass effect, and treatment plans are personalized based on assessments of the prognosis with and without surgical therapy. In our patient, craniotomy with tubular evacuation of the hematoma led to improvement in the symptoms and prevented the potential life-threatening herniation sequelae based on clinical examination.

In the International Surgical Trial in IntraCerebral Hemorrhage (STICH) trial of 1033 patients with supratentorial ICH, those who had early surgical hematoma evacuation had similar favorable outcome rates as those who received maximum medical therapy [17]. A trend toward favorable outcomes was observed among patients assigned to early surgery who had craniotomy as opposed to alternate techniques and in those with hematoma located 1 cm or less from the cortical surface. In the STICH II trial when comparing patients who received surgery to conservative care, the unfavorable functional outcome was similar at six months. However, individuals who received early surgery showed a trend toward lower mortality [3]. Surgery was linked to lower mortality and a trend toward improved functional outcomes was observed in a systematic review that included 15 trials with more than 3000 patients with SICH, the benefit was greatest for people with worse prognoses at the time of presentation, people who got worse after they presented, and people who had superficial ICH without intraventricular extension [18].

Supratentorial hematoma evacuation may decrease mortality in patients who are unconscious, have a big hematoma( thickness greater than 10 mm or a midline shift greater than 5 mm on computed tomographic (CT) scan) with a major midline shift, or have excessive ICP that is resistant to medical therapy [16]. Although it may lower mortality, supratentorial decompression with hematoma evacuation and/or decompressive hemicraniectomy does not necessarily lead to better functional outcomes [17]. Even in these situations, judgments should be made individually. It should only be regarded as a life-saving operation to treat resistant increases in ICP.

The most extensively researched surgical procedure for patients with supratentorial ICH is open craniotomy with craniectomy. Other procedures involving a craniotomy include minimally invasive surgical interventions like CT-guided stereotactic aspiration, endoscopic hemorrhage aspiration, and fibrinolytic treatment to remove the hematoma before aspiration [19]. Minimally invasive surgical intervention is selected in some cases to address symptoms, large hemorrhage volume with midline shift, and peri-hemorrhagic brain edema with a primary goal to decrease mortality over functional outcomes. Management is still controversial between surgical and medical arms with more data about survival in minimally invasive brain approaches [20]

## Conclusions

SICH involving the temporal lobe and concomitant with SAH and SDH is a rare entity. However, clinicians should be cautious as SICH can occur without hypertension, trauma, or ruptured AVM. With debatable benefits between conservative and surgical intervention, judgments should be made individually. Surgical evacuation of the hematoma should only be regarded as a life-saving operation to treat resistant increases in ICP. In all cases, conducting a comprehensive evaluation is essential, as it can assess the presence of intracerebral hemorrhage or surrounding swelling, which may impede and complicate the assessment of any underlying structural abnormalities that could be responsible for SICH*.*
